# A Novel Method for Breath Detection via Stepped-Frequency Continuous Wave Ultra-Wideband (SFCW UWB) Radars Based on Operational Bandwidth Segmentation

**DOI:** 10.3390/s18113873

**Published:** 2018-11-10

**Authors:** Hao Lv, Teng Jiao, Yang Zhang, Fulai Liang, Fugui Qi, Jianqi Wang

**Affiliations:** Department of Medical Electronics, School of Biomedical Engineering, Fourth Military Medical University, Xi’an 710032, China; fmmulvhao@126.com (H.L.); jiaoteng@fmmu.edu.cn (T.J.); yangzhang@fmmu.edu.cn (Y.Z.); liangfulai@fmmu.edu.cn (F.L.); qifgbme@outlook.com (F.Q.)

**Keywords:** UWB radar, human being, breath detection, operational bandwidth, segmentation

## Abstract

Human being detection via ultra-wideband (UWB) radars has shown great prospects in many areas, such as biomedicine, military operation, public security, emergency rescue, and so on. When a person stays stationary, the main feature that separates him/her from surroundings is the movement of chest wall due to breath. There have been many algorithms developed for breath detection while using UWB radars. However, those algorithms were almost based on a basic scheme that focused on processing in the time dimension of UWB data. They did not utilize the benefits from the wide operational bandwidth of UWB radars to show potential superiority over those narrowband systems such as a continuous wave (CW) Doppler radar. In this paper, a breath detection method was proposed based on operational bandwidth segmentation. A basic theoretical model was firstly introduced, indicating that characteristics of breath signals contained in UWB echoes were consistent among the operational frequencies, while those of clutters were not. So, the method divided a set of UWB echo data into a number of subsets, each of which corresponded to a sub-band within the operational bandwidth of the UWB radar. Thus information about the operational frequency is provided for subsequent processing. With the aid of the information, a breath enhancement algorithm was developed mainly by averaging the segmented UWB data along the operational frequency. The algorithm’s performance was verified by data measured by a stepped-frequency CW (SFCW) UWB radar. The experimental results showed that the algorithm performed better than that without the segmentation. They also showed its feasibility for fast detection of breath based on a short duration of data. Moreover, the method’s potential for target identification and impulse-radio (IR) UWB radar was investigated. In summary, the method provides a new processing scheme for UWB radars when they are used for breath detection. With this scheme, the UWB radars have a benefit of greater flexibility in data processing over those narrowband radars, and thus will perform more effectively and efficiently in practical applications.

## 1. Introduction

The technology of human being detection via radars has caused great concern in recent years, since it can be applied in many areas like biomedicine, military operation, public security, emergency rescue, and so on [1,2,3,4,5,6,7,8,9,10,11]. There have been two major kinds of radar systems used for the technology: one is narrowband radars represented by the continuous wave (CW) Doppler system and the other is ultra-wideband (UWB) radars. According to the FCC rules, a UWB radar is one that has a relative bandwidth larger than 25% or an absolute operational bandwidth larger than 500 MHz. Compared with CW Doppler radars, UWB radars have many benefits for human being detection due to the wide operational bandwidth, for instance, improved range resolution, greater penetration capability, better electromagnetic compatibility, etc. [12,13,14,15,16,17,18,19,20,21,22,23,24,25,26]. Among UWB radars, the most frequently used is the impulse-radio (IR) UWB radar [12,13,14,15,16,17,18,19,20,21,22]. An IR UWB radar transmits pulses with very short durations in the nanosecond range or even less to achieve an operational bandwidth in the hundreds of MHz, with benefits for human being detection, particularly in non-line-of-sight scenarios, such as searching buried victims in post-earthquake scenario and tracking moving suspects in through-wall surveillance [12,13,14,15]. In these scenarios, the main feature that separates a human being from surroundings is the movement of chest wall due to breath when he or she is stationary. Since displacement of the movement is on the order of millimeters and great amounts of clutters caused by scattering from the surroundings, an algorithm should be developed to process UWB data for breath detection. Up to now, many algorithms have been developed for this purpose [12,13,14,15,16,17,18,19,20,21,22]. For example, a basic processing scheme was proposed for respiration detection via an IR UWB radar in [12], in which a motion filter was utilized to remove the stationary background clutters and a fast Fourier transform (FFT) followed in the time dimension of the data to estimate the spectrum of respiration. Based on the scheme, the algorithms in [13,14,15] were designed not only for breath detection but also for target identification particularly for post-earthquake searching and rescuing operations. Especially, in [14], the breath’s periodicity or quasi-periodicity characteristic in time was exploited by an adaptive line enhancer. In [15], the Hilbert–Huang transform (HHT) was used for the time–frequency analysis to separate breathing signals from two human subjects. To further improve the resolution of the estimated spectrum, a Chirp Z-Transform (CZT) was applied instead of FFT for breath and heartbeat monitoring [16]. To deal with the harmonics and inter-modulation products that plague signal resolution in widely used FFT spectrograms, a super-resolution spectral algorithm that is based on the state space method (SSM) was developed in [17]. Moreover, a breath detection algorithm was proposed based on the multichannel singular spectrum analysis (MSSA) technique for the suppression of the non-stationary noises and clutters [18] and the hidden Markov model or the Kalman model was used for the body orientation issue during breath detection [19,20], and an exponential moving averaging filter was used for suppressing the clutters that are caused by the body movements [21], and a phase method that previously used for CW radars was proposed for accurate heartbeat extraction via UWB radars [22]. Except for IR UWB radars, stepped-frequency CW (SFCW) UWB radars, pseudo-random UWB radars, and so on, which have been claimed to possess advantages over IR UWB ones, have also been reported to detect human beings’ breath [23,24,25,26]. However, there’s no essential difference in the corresponding algorithms with those used by IR UWB radars.

All the above breath detection algorithms used by UWB radars almost focused on processing in the time dimension of UWB data. The same scheme can also be used for a CW Doppler radar. It is worth noting that a UWB radar provides us much more benefits by its ultra-wide operational bandwidth than a CW Doppler radar. If this is utilized properly in data processing, improved detection performance could be achieved very likely. However, no literature is reported to this problem in traditional UWB-radar-based breath detection algorithms, which ends up with a similar detection performance with a CW Doppler radar. Therefore, attempting to fix this gap, a novel breath detection method was proposed by investigating and exploiting the ultra-wide operational bandwidth of UWB radars. The method firstly divided UWB data into a number of subsets, each of which corresponded to a sub-band of the operational bandwidth. Thus the UWB data were added with the operational frequency information (the operational frequency was used in this paper to make a difference with the frequency information that is obtained by performing FFT along the time dimension of UWB data). Then, by averaging along the additional operational frequency, a breath enhancement algorithm was developed. Lastly, the algorithm’s performance was verified using data measured by a SFCW UWB radar. The results showed that the algorithm performed effectively on those data. Besides, the method’s feasibilities for human target identification and IR UWB radar were investigated.

The rest of the paper are organized, as following: Section 2 introduces a basic theoretical model that represents the UWB echoes in the operational frequency domain; Section 3 describes the experiment setup and method, including the SFCW UWB radar and the breath enhancement algorithm; Section 4 presents the experimental results; Discussion and conclusions are drawn in Section 5.

## 2. Basic Theoretical Model

### 2.1. Model Description and Analysis

Consider a UWB radar with a single transmitter and a single receiver that are configured in a monostatic scheme. Let the transmitter transmit a UWB signal s(t). For the case of a single human being, it can be simplified as a point target located in range d. When the target stands motionlessly and his/her breath is the only concern, the range of the target can be expressed as $d=d_{0}+\Delta dsin\left(2\pi f_{b}t\right)$, where $d_{0}$ is the target’s nominal range and $\Delta dsin\left(2\pi f_{0}t\right)$ is the range variation of the chest wall due to breath that’s modeled as a sine function with Δd denoting the breathing amplitude and $f_{b}$ the breathing frequency. In UWB echoes, the range corresponds to a fast time delay τ=2d/c, and is given by

(1) $$\tau =\tau_{0}+\Delta \tau sin\left(2\pi f_{b}t\right)$$

where $\tau_{0}=2d_{0}/c$, Δτ=2Δd/c, and c is the speed of light. Thus the echoes, neglecting propagation loss and distortion, can be given by

(2) r(t)=αs(t−τ)

where α is the complex target reflectivity that can be assumed independent on frequency for simplicity. By denoting the Fourier transform of s(t) as S(ω), the operational-frequency-domain representation of the received signals can be expressed as

(3) R(ω)=αS(ω)exp(−jωτ)

where ω represents the operational frequency in radians. By combining Equations (1) and (3),

(4) $$R\left(\omega ,t\right)=\alpha S\left(\omega \right)\exp\left(-j\omega \tau_{0}\right)\exp\left[-j\omega \Delta \tau \sin\left(2\pi f_{b}t\right)\right]$$

This is the basic theoretical model for breath detection via UWB radars. The received signals are two-dimensional with information about the operational frequency ω and the time t. The breath signal represents itself in them as a phase shift of the transmitted signals. Besides, the operational frequency can further be transformed into range by an inverse FFT (IFFT) [3]

(5) $$r\left(\tau ,t\right)=\frac{\alpha }{2\pi j}\overset{+\infty }{\underset{-\infty }{\int }}\;R\left(\omega ,t\right)\exp\left(j\omega t\right)d\omega$$

After that, the received signals become two-dimensional with range τ and time t.

Firstly, consider a non-breathing case, in which Equation (4) can be viewed as a clutter model for breath detection and becomes

(6) $$R\left(\omega \right)=\alpha S\left(\omega \right)\exp\left(-j\omega \tau_{0}\right)$$

It implies that the clutters are dependent on S(ω) [3]. Now, when considering the propagation loss and distortion, S(ω) should be replaced by an attenuated and distorted version of the transmitted signals’ Fourier transform. In practice, as the signals propagate through obstacles, such as walls and building ruins whereby the dielectric characteristics and structures are usually unknown in priori, S(ω) is not deterministic. Thus, the clutters can be viewed as random among the operational frequencies.

Then consider Equation (4) in a breathing case. The expression $\exp\left[-j\omega \Delta \tau \sin\left(2\pi f_{0}t\right)\right]$ in the integral can be expanded by Fourier series [4]

(7) $$\exp\left[-j\omega \Delta \tau \sin\left(2\pi f_{b}t\right)\right]=\overset{+\infty }{\underset{n=-\infty }{\sum }}J_{n}\left(\Delta \tau \right)\exp\left(jn2\pi f_{b}t\right)$$

where $J_{n}\left(x\right)$ is the *n*th-order Bessel function of the first kind. By introducing the Bessel function, which is regular with time t, the breath signals contained in R(ω,t) are expected to be consistent among operational frequencies.

### 2.2. Model Simulation and Results

To investigate the above analysis, the basic theoretical model was simulated via a stepped-frequency synthesization. Firstly, the discrete form of Equation (4) is given, as follows

(8) $$R\left[m,n\right]=\alpha S\left[2\pi \left(f_{0}+m\Delta f\right)\right]\exp\left[-j2\pi \left(f_{0}+m\Delta f\right)\tau_{0}\right]\exp\left[-j2\pi \left(f_{0}+m\Delta f\right)\Delta \tau \sin\left(2\pi f_{b}nT_{s}\right)\right]$$

where m=0,1,…,M−1, n=0,1,…, N−1; ω=2πf, and $f_{0}$, Δf, $T_{s}$ denotes the starting point, step size, sampling period of the stepped-frequency signal, respectively. Thus the received signals, namely the echo data, can be viewed as a two-dimensional (operational frequency and time) M×N matrix. In the above equation, M denotes the sampling number in the operational frequency. Thus the operational bandwidth is determined as (M−1)Δf. N denotes the sampling number in the time. Together with the sampling period $T_{s}$, the time duration of the echo matrix is $\left(N-1\right)T_{s}$. For convenience of the subsequent analysis, the discrete form of Equation (5) is also given below

(9) $$r\left[m,n\right]=\frac{\alpha }{2\pi j}\overset{f_{0}+m\Delta f}{\underset{f=f_{0}}{\sum }}R\left[m,n\right]\exp\left[j2\pi \left(f_{0}+m\Delta f\right)nT_{s}\right]$$

where m=0,1,…,M−1, n=0,1,…, N−1. r[m,n] is also a two-dimensional M×N matrix but with M denoting the sampling number in the range. In the range dimension, the unambiguous range is determined by the step size as c/(2Δf).

**Figure 1:**
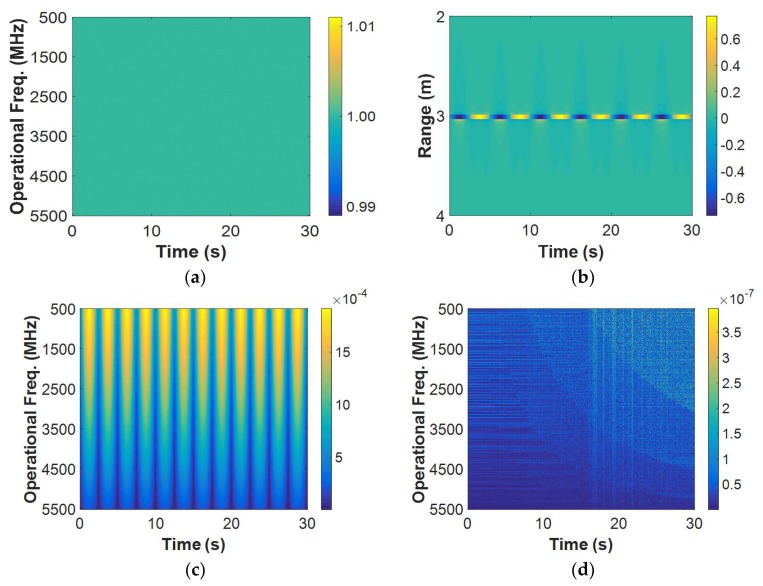
Model simulation results: (**a**) echo matrix in a breathing case; (**b**) result matrix after the background removal in a breathing case; (**c**) result matrix after the inverse fast Fourier transform (IFFT) on range in a breathing case; and, (**d**) result matrix after the IFFT on range in a non-breathing case.

Figure 1 shows the simulated results of the model. During the simulation, $f_{0}=500\;\mathrm{MHz},\Delta f=10\;\mathrm{MHz},\;T_{s}=0.005s,\;M=501,\;N=2001$ for the stepped-frequency signal. It means that the operational bandwidth is 5000 MHz, within which $S\left(f_{0}+m\Delta f\right)$ was uniformly set. It also means that the unambiguous range is 15 m and the time duration is 30 s. For the target, $\alpha =1,\;d_{0}=3\;m,\;\Delta d=0.005\;m,\;f_{b}=0.2\;\mathrm{Hz}$. Figure 1a shows the echo data matrix R[m,n] in Equation (8). The matrices are actually complex and shown herein by getting the absolute values. In this figure, neither characteristics of the breath signals nor those of the clutters can be clearly seen. So the IFFT was performed on the operational frequency, as in Equation (9), to get the matrix r[m,n]. Then, a background removal was performed to r[m,n], so as to remove the static component that is too strong to disguise the breath signals [14]. It was realized by subtracting the mean from each row in r[m,n], namely $r\left[m,n\right]-1/\left(N-1\right)\overset{N-1}{\underset{n=1}{\sum }}r\left[m,n\right]$. The result matrix after that is shown in Figure 1b, in which only the data between 2 m and 4 m were shown for clearness. Periodically varying strips along the time can be clearly observed at the range of 3 m in this figure. These are breath signals in accordance with the target settings. Since the result matrix only contains breath signals, IFFT was performed on its range dimension to get the operational frequency information again. Thus, the characteristics of the breath signals among the operational frequencies can be investigated. The result matrix after that is shown in Figure 1c. In this figure, vertical strips can be clearly seen along the operational frequency dimension of the result matrix. It indicates a consistency along the dimension for the breath signals. For comparison, a non-breathing case was also simulated according to the discrete form of Equation (6). The echo matrix in the case was also processed as that in the breathing case, and the result matrix after the IFFT on range is shown in Figure 1d. No regular strips can be seen this figure. As the non-breathing case can be viewed as the clutter model, it implies that there’ is no consistency among the operational frequencies for clutters.

So, it has been evident that the breath signals and clutters have different characteristics in the operational frequency. Using this kind of difference, data processing algorithms can be developed to improve the performance of UWB radars for breath detection. An intuitive way for this is to average UWB data in the dimension of operational frequency. Breath signals are expected to be enhanced due to their consistency among the operational frequencies, while clutters or noises are not.

## 3. Experiment Setup and Method

### 3.1. SFCW UWB Radar

**Figure 2:**
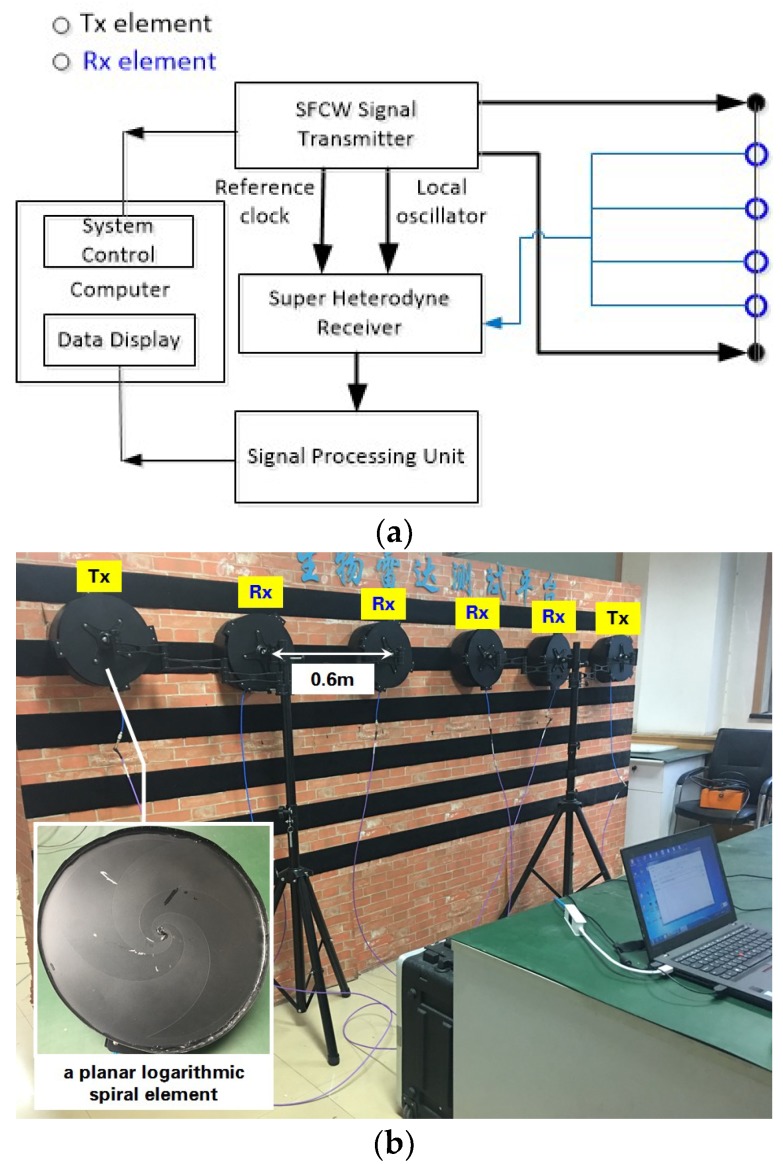
(**a**) Block diagram of the Stepped-Frequency Continuous Wave Ultra-Wideband (SFCW UWB) radar [25] and (**b**) experiment setup.

To verify the above analysis, a MIMO (Multiple Input and Multiple Output) SFCW UWB radar was used for experiments. The block diagram of the radar and the experimental setup are shown in Figure 2. The radar had two transmitting and four receiving antennas, all in the form of planar logarithmic spiral elements, to have eight data channels. In the experiments, the antennas were configured into a uniform line array that closely clung to surface of an approximately 30-cm-thick brick wall, and a healthy male of 25 years stood behind the wall facing the radar as a target [25]. The key parameters of the radar are listed in Table 1. Note that the 40–4400 MHz operational frequency means that the radar’s bandwidth is 4360 MHz. Not aiming at human being localization or imaging, echo data only from the 8th channel, i.e., from the No. 2 transmitting element to the No. 4 receiving element, was used. The echo data were stored in the matrix R[m,n]. The sampling number in the operational frequency M was 874 and that in the time N depended on each measurement. All of the data were post-processed in the MATLAB environment on the computer, including the simulation in Section 2.

### 3.2. Breath Enhancement Algorithm

#### 3.2.1. Operational Bandwidth Segmentation

**Figure 3:**
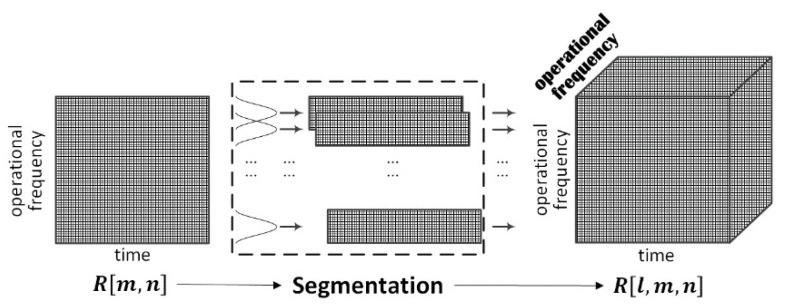
Diagram of the operational bandwidth segmentation.

To get information about the operational frequency, an operational bandwidth segmentation method was designed firstly. The method sampled the echo matrix R[m,n] into a number of sub-bands, instead of individual operational frequencies to remain a certain range resolution, by a window moving along the operational frequency dimension of the matrix. It can be expressed as follows

(10) $$\begin{array}{c} R\left[l,m,n\right]=R\left[m,n\right]\cdot \mathrm{rect}\left[l\right]\;\mathrm{with}\;\mathrm{rect}\left[l\right]=\{\begin{array}{l} 1,\;l\Delta l+1\leq m\leq l\Delta l+L\\ 0,\;otherwise \end{array},\\ l=0,1,\ldots \left(M-L+1\right)/\Delta l,\;m=0,1,\ldots ,M-1,\;n=0,1,\ldots ,\;N-1 \end{array}$$

where L and Δl denotes the window length and the moving step of the window, and ⎣·⎦ represents getting modulus. The window’s function rect[l] equals to 1 when lΔl+1≤m≤lΔl+L, namely within the sub-band from (lΔl+1)Δf to (lΔl+L)Δf, while it equals to 0 otherwise. Thus the echo matrix was divided into ⎣(M−L+1)/Δl⎦+1 sub-matrices corresponding to different sub-bands. Then the sub-matrices were stored together to form a three-dimensional result matrix R[l,m,n]. The diagram of the procedure is shown in Figure 3. It indicates that a new dimension, namely l, is added to the echo matrix R[m,n]. Note that the dimension also contains information about the operational frequency. So, the result matrix has two dimensions of operational frequency. One corresponds to m that is original in the R[m,n]. The other, in the bold font, is the additional one l.

#### 3.2.2. Basic Processing Flow

**Figure 4:**
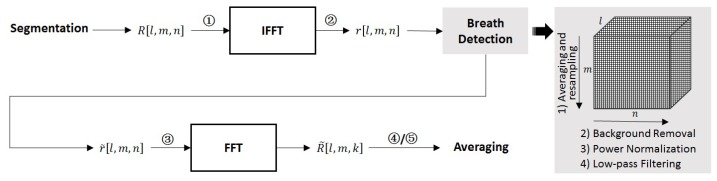
Basic processing flow of the breath enhancement algorithm.

Figure 4 shows the basic processing flow of the breath enhancement algorithm. The result matrix after the segmentation, namely R[l,m,n], was first processed by IFFT along its original operational frequency m. As similar in Equation (9), this step transformed the operational frequency into the range and it gets the three-dimensional (operational frequency, range, and time) matrix r[l,m,n]. It can be expressed, as follows

(11) $$r\left[l,m,n\right]=\frac{\alpha }{2\pi j}\overset{f_{0}+m\Delta f}{\underset{f=f_{0}}{\sum }}R\left[l,m,n\right]\exp\left[j2\pi \left(f_{0}+m\Delta f\right)nT_{s}\right]$$

Then, breath detection was performed [18,20]. In turn, it includes: (1) averaging and resampling on the range to improve the SNCR (signal-to-noise-and-clutter ratio) and reduce the sampling number; (2) a 160-order FIR (Finite Impulse Response) motion filter, implemented by moving average subtraction on the time, to remove the backgrounds due to the strong scatterings from those statistic objects such as the wall; (3) power normalization on the time, namely transforming the numerical variation along the dimension into the scope [–1, 1], to balance the propagation attenuation among the ranges; and, (4) a 321-order FIR low-pass filter with the cutoff frequency of 0.5 Hz on the time dimension to detect out the breath signals. After that, the result matrix (denoted as $\tilde{r}\left[l,m,n\right]$) was finally processed by FFT on its time to get the three-dimensional (operational frequency, range, and frequency) matrix $\tilde{R}\left[l,m,k\right]$.

#### 3.2.3. Averaging along the Operational Frequency

In the above flow, the UWB data were processed orderly in a three-dimensional manner. Thus the averaging along the operational frequency can be performed during different phases in the flow. The following cases were considered: ① to average R[l,m,n], namely the result matrix after the segmentation and with the original operational frequency, which is denoted as AoF; ② to average r[l,m,n], namely the result matrix after the IFFT with the range, which is denoted as AoR; ③ to average $\tilde{r}\left[l,m,n\right]$, namely the result matrix after the breath detection and with the time information, which is denoted as AaT; ④ to average $\tilde{R}\left[l,m,k\right]$, namely the result matrix after the FFT on time and with the frequency information, which is denoted as AaF; and, ⑤ averaging of $\mathrm{abs}\left(\tilde{R}\left[l,m,k\right]\right)$, namely the result matrix after getting the absolute values of $\tilde{R}\left[l,m,k\right]$, which is denoted as AaA. The process can be expressed as

(12) $$\tilde{R}\left[m,k\right]=\overset{\left(M-L+1\right)/\Delta l}{\underset{l=1}{\sum }}X\left[l,m,n/k\right]$$

where X[l,m,n/k] can be replaced with R[l,m,n], r[l,m,n], $\tilde{r}\left[l,m,n\right]$, $\tilde{R}\left[l,m,k\right]$, or $\mathrm{abs}\left(\tilde{R}\left[l,m,k\right]\right)$. The result matrix $\tilde{R}\left[m,k\right]$ is a two-dimensional matrix with range and frequency again. Thus a power peak would appear in the result matrix, when there is a human being in the scene, with the peak locating at the human being’s range and breathing frequency [20].

## 4. Experimental Results

### 4.1. Algorithm Performance Analysis

#### 4.1.1. Example Results

**Figure 5:**
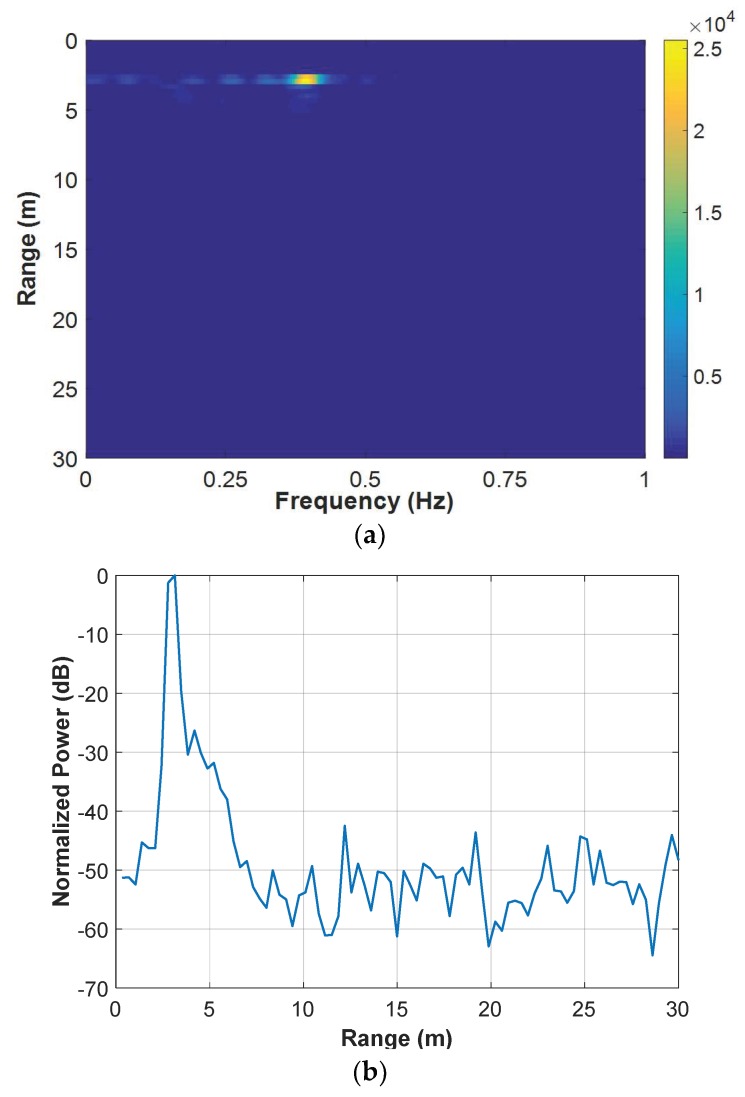
Example results of a set of data measured by the SFCW UWB radar with the human target being 3 m behind the wall: (**a**) result matrix after the breath enhancement and (**b**) its power-range plot.

Figure 5 shows the example results of a set of data measured by the SFCW UWB radar, with the human target being 3 m behind the wall. The window length L and the moving step Δl during the segmentation was 100 and 10, respectively. So, the echo matrix R[m,n] was divided into 78 sub-matrices to form the three-dimensional matrix R[l,m,n]. During the breath enhancement, or to be more precisely, by the averaging and resampling step in the breath detection, the sampling number in the range was down-sampled from 874 to 86. Then, the averaging along the operational frequency was performed in the AaT case, namely $X\left[l,m,n\right]=\tilde{r}\left[l,m,n\right]$ in Equation (12). Finally, the FFT was performed in the time dimension to result in the two-dimensional matrix $\tilde{R}\left[m,k\right]$. The result matrix is shown in Figure 4a. The power peak due to the target’s breath can clearly be observed in the figure. It indicates that the target’s range is approximately 3 m and his breathing frequency is approximately 0.39 Hz. For the convenience of subsequent analysis, a power-range plot was calculated by picking the maximum value from each row of the matrix. It can be expressed as

(13) $$\begin{array}{l} \tilde{R}\left[m\right]=\underset{k}{\max}\tilde{R}\left[m,k\right]\\ \tilde{R}\left[m\right]=20\log_{10}\left(\tilde{R}\left[m\right]/\max\left(\tilde{R}\left[m\right]\right)\right) \end{array}$$

where $\tilde{R}\left[m\right]$ represents the power-range plot and is shown in Figure 4b. A power peak due to the breath can also be observed at the corresponding range in the plot. Note that it is logarithmically normalized with its maximum value being 0 dB. Thus, the SNCR can be easily evaluated by observing the power floor in the plot. It is roughly 50 dB for this figure.

#### 4.1.2. Effects of the Segmentation Parameters

**Figure 6:**
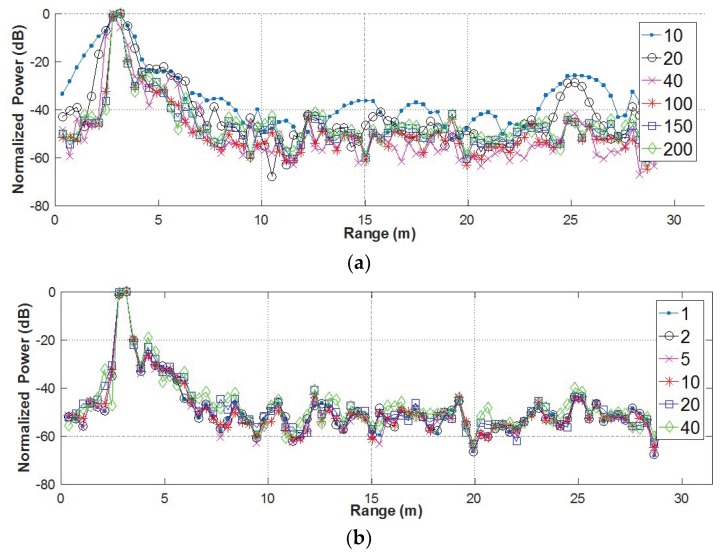
Power-range plots for (**a**) different window sizes and (**b**) different moving steps in the operational bandwidth segmentation.

Two parameters, namely the window length L and the moving step Δl, were included in the operational bandwidth segmentation. Based on the above data, their effects on the breath enhancement performance were investigated. The averaging was also performed in the AaT case. Firstly, L varied from 10 to 200, with Δl being fixed to 10. The corresponding normalized power-range plots are shown in Figure 6a. The figure indicates that the SNCR’s do not vary much among those window sizes. However, an improvement of range resolution can be roughly observed, by the width of the peaks in the figure, as the window size becomes larger. This is due to the fact that the window size determines the bandwidth of the segmented sub-bands and thus the range resolution. But, the range resolution does not improve when the window size is larger than 100. Then, the effect of Δl was investigated by varying the parameter from 1 to 40 with L=100. As shown in Figure 6b, there is no significant difference among the power-range plots.

#### 4.1.3. Effect of the Different Averaging Cases

**Figure 7:**
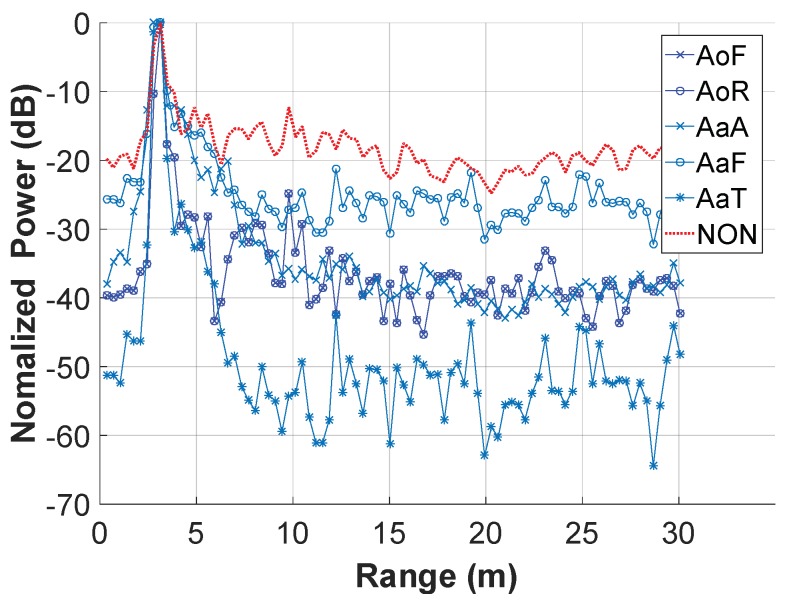
Power-range plots in different averaging cases during the breath enhancement algorithm.

Figure 7 shows the power-range plots for different averaging cases along the operational frequency. They were calculated with the segmentation parameters L=100, Δl=10. Note that NON in this figure represents the plot calculated from the result matrix that was processed without the segmentation and the averaging. It was actually a two-dimensional version of the flow in Figure 4. In Figure 7, the SNCR’s are roughly 20 dB, 30 dB, 40 dB, 40 dB, 40 dB, and 50 dB for NON, AoF, AoR, AaA, AaF, and AaT, respectively. It indicates that the breath enhancement algorithm performed effective in all the averaging cases but best in the AaT case. This might be resulted from two aspects: one is that in the AaT case the algorithm averaged the data with the most degrees of freedom, and the other lies in the breath detection that improved the consistency of the breath signals among the operational frequencies.

### 4.2. Breath Enhancement Results

**Figure 8:**
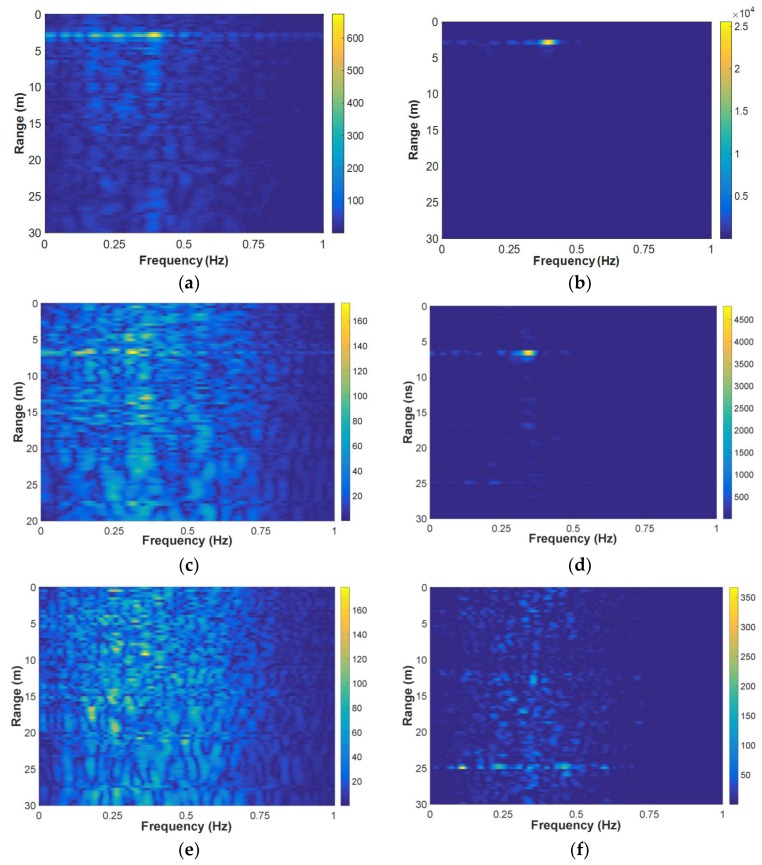
Result matrices: (**a**) processed without the segmentation and averaging when the human target stood 3 m behind the wall; (**b**) processed with the breath enhancement algorithm when the human target stood 3 m behind the wall; (**c**) processed without the segmentation and averaging when the human target stood 7 m behind the wall; (**d**) processed with the breath enhancement algorithm when the human target stood 7 m behind the wall; (**e**) processed without the segmentation and averaging when the human target stood 9 m behind the wall; and, (**f**) processed with the breath enhancement algorithm when the human target stood 9 m behind the wall.

Figure 8 shows the results of three sets of data measured by the SFCW UWB radar, with the human target 3 m, 7 m, and 9 m behind the wall, respectively. The data were all segmented with L=100, Δl=10, and averaged in the AaT case. For comparison, the result matrices corresponding to the NON case are also shown. Obviously in the Figure 8a–d, the SNCR’s are much higher for the enhancement algorithm than those for the NON case. But, for the 9 m data, namely the target stood approximately 9m behind the wall, the result matrices in Figure 8e,f show that neither the two algorithms performed effectively. This may be due to that the echoes from the target became too weak to be detected by the radar in such a scenario. But, at any rate, the algorithm performed more effectively than that without the segmentation and averaging for the same data.

**Figure 9:**
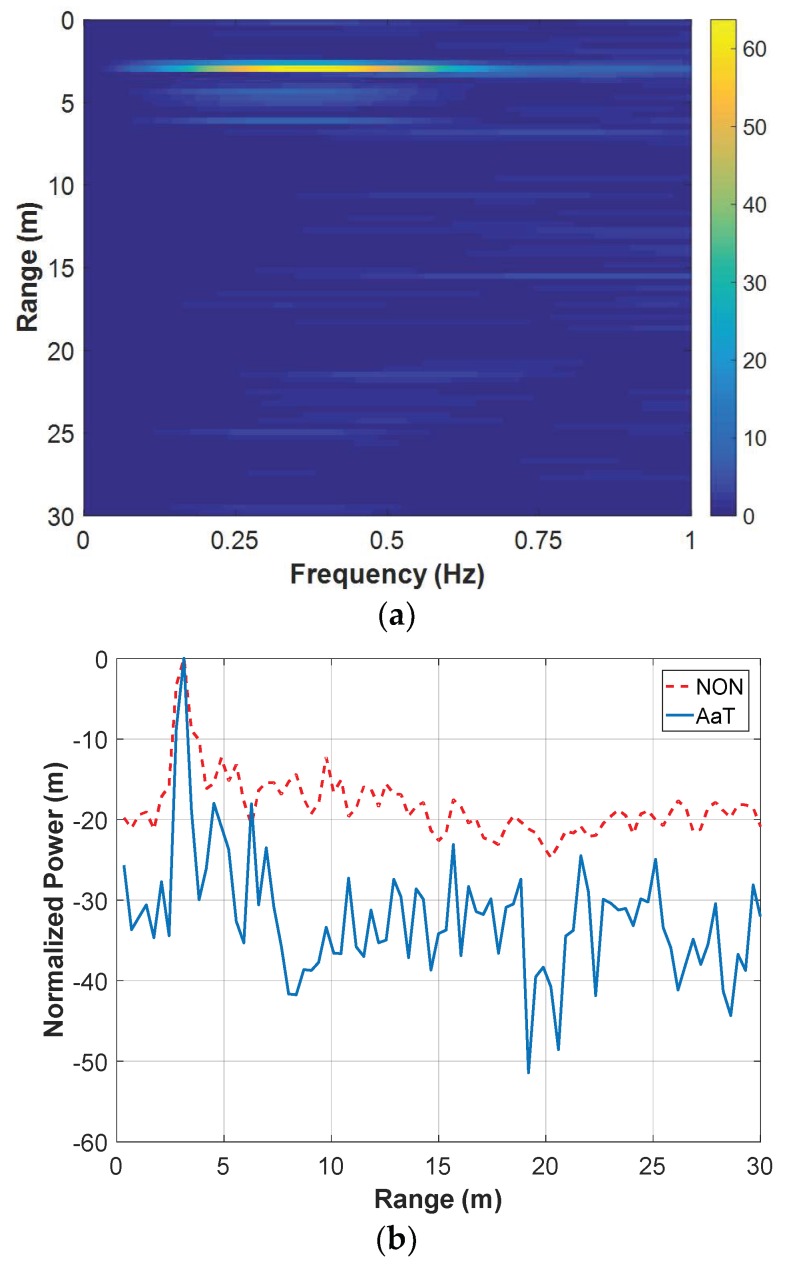
Results of the data measured by the SFCW UWB radar with the human target being 3 m behind the wall: (**a**) result matrix after the breath enhancement for the first 2 s and (**b**) its power-range plot.

Figure 9 shows the results corresponding to the set of data measured when the human target stood 3 m behind the wall. Only the first 2 s were processed by the breath enhancement algorithm. Limited by the time duration, the background removal step used the mean subtraction method (as in the model simulation) instead of the moving average subtraction, and the 321-order FIR low-pass filter was omitted. Figure 9a shows the result matrix, in which the power peak due the breath can be clearly seen, although the frequency resolution is quite poor for accurately evaluating the breathing frequency. Figure 9b shows the power-range plot. For comparison, the power-range plot corresponding to the NON (identical to that in Figure 7) case was also shown. It indicates that, even for such a short duration of data, the breath enhancement algorithm results in a SNCR higher than that of the NON case.

### 4.3. Target Identification Results

**Figure 10:**
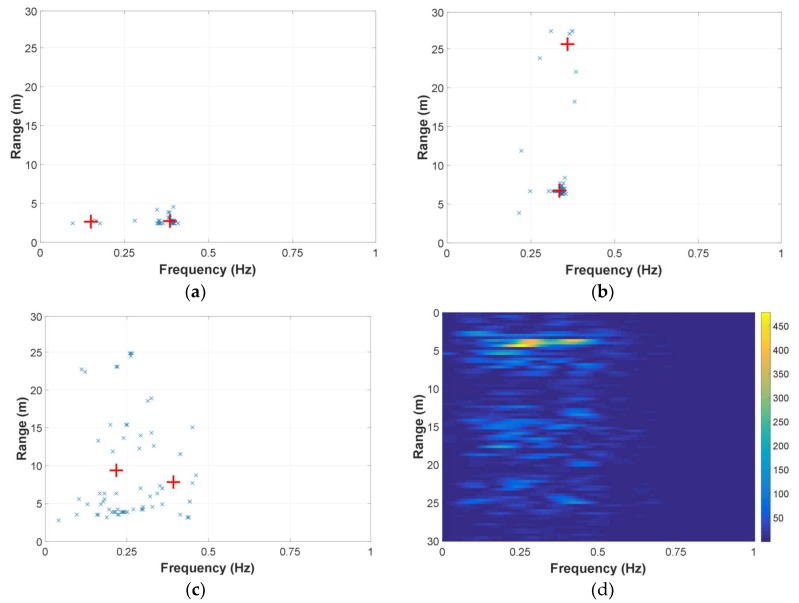
(**a**) Power peaks of the sub-matrices and clustering result for the data measured with the human target being 3 m behind the wall; (**b**) Power peaks of the sub-matrices and clustering result for the data measured with the human target being 7 m behind the wall; (**c**) Power peaks of the sub-matrices and clustering result for the data without human target; and, (**d**) result matrix after the breath enhancement for the data without human target.

Except for the breath enhancement, the feasibility of the operational bandwidth segmentation for target identification was also preliminarily investigated. See the processing flow in Figure 4, the three-dimensional (operational frequency, range and frequency) $\tilde{R}\left[l,m,k\right]$ can also be viewed as a number of two-dimensional range-frequency matrices. Only considering the one-target case, the power peaks of the matrices were picked up individually and put together. Then a k-means clustering, where k was set to 2 considering the target and those outliers caused by clutters, was used to cluster those peaks to identify the target. Three sets of data measured by the SFCW UWB radar were used for performance demonstration, namely those measured when the human target were 3 m and 7 m away behind the wall and that measured with no human target. The clustering results are shown in Figure 10, in which the two centroids resulted from the clustering are marked as red solid crosses. For the case of the human target being 3 m away, as depicted in Figure 10a, the right one of the two centroids locates in accordance with the peak location of the result matrix after the enhancement, as depicted in Figure 8b. It is the same with the case of the human target 7 m away by comparing Figure 10b and Figure 8d. But, for the data with no human target, both the locations of the two centroids in Figure 10c are neither in accordance with those in Figure 10d. In this way, a false alarm can be avoided for the non-target case.

### 4.4. IR UWB Radar Results 

**Figure 11:**
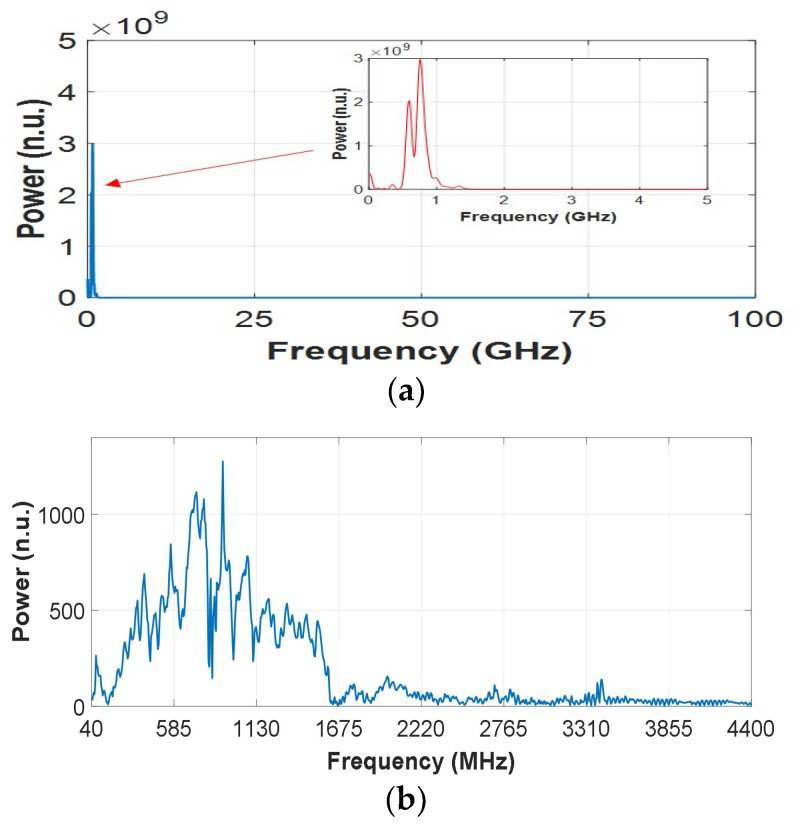
Cases of the echo spectrum (**a**) of the impulse-radio (IR) UWB radar and (**b**) of the SFCW UWB radar.

Except for the SFCW UWB radar, a set of data previously measured by an IR UWB radar was used to verify the breath enhancement algorithm. The parameters of the radar can be referred to in [20], by which the data were also measured in a through-wall scenario. Since the echo data of an IR UWB radar are two-dimensional range-time ones, the operational bandwidth segmentation was implemented by windowing the FFT results on the data’s range dimension and then performing IFFT back to the range-time domains. Besides, the window size and moving step were different from those that were used by the SFCW UWB radar. The result matrix showed no SNCR improvement compared with that processed without the segmentation and averaging. It might be caused by the characteristics of the echo spectrum of the IR UWB radar. A case of the spectrum, together with that of the above SFCW UWB radar, were shown in Figure 11. As in the figure, the IR UWB echo spectrum’s energy concentrates on a very small section compared with the large frequency span, which results in a 400 MHz operational bandwidth of the radar. This is caused by a very high sampling frequency (approximately 100 GHz) due to the equivalent-time sampling architecture by most of IR UWB radars [12,13,14,15,16,17,18,19,20,21,22]. Thus valid information can hardly be provided by segmenting the operational bandwidth into different frequencies or sub-bands. However, the SFCW UWB echo spectrum’s energy roughly overspread the operational bandwidth. It makes the operational bandwidth operation and the breath enhancement feasible. Note that the spectrum of the SFCW UWB radar presents a significant suppression in the high-frequency bands (approximately from 1600 MHz to 4400 MHz), and obvious frequency notches in the low-frequency bands (below 1600 MHz). They are mainly due to the effects, such as attenuation, multipath, etc., when the electromagnetic waves propagate through the wall.

## 5. Discussion and Conclusions

A novel method based on operational bandwidth segmentation was proposed for breath detection via UWB radars in this paper. First of all, a basic theoretical model was introduced and simulated to represent UWB radar echoes in the operational frequency domain. It indicated that the characteristics of breath signals that are contained in UWB echoes were consistent among operational frequencies while clutters were not. So, the difference was used in data processing to improve breath detection by averaging along the operational frequency. To verify this, a SFCW UWB radar was used for experiments and a breath enhancement algorithm was developed. The radar worked with an operational bandwidth of 40–4400 MHz and measured the breath of a human target behind a brick wall. The algorithm consisted of an operational bandwidth segmentation, a basic processing flow and an averaging along the operational frequency. The segmentation divided the two-dimensional UWB echo data into a number of subsets by applying a moving window along the operational frequency dimension of the data. Since each subset corresponded to a sub-band of the operational bandwidth, information about the operational frequency was added to the echo data for the subsequent processing and averaging. The experimental results firstly showed the algorithm’s performance with different segmentation parameters, namely the window length and moving step of the moving window, as well as that in different cases of averaging. With the parameters and the case that had the best performance, the algorithm was verified by different sets of data measured by the SFCW radar. The results showed a significant improvement of SNCR that was detected by the algorithm than by the basic processing flow that had neither segmentation nor averaging. Thus, it can be used to improve the effectiveness of the radar in practical applications that need breath detection. The results also showed that the algorithm’s effectiveness based on a short duration of data. So, it can avoid the coherent accumulation (e.g., in the form of FFT on the time) on a long duration (at least one or two breathing cycles) of data, just as that in the existing breath detection algorithms. This implies the feasibility of a fast detection of breath, which is surely helpful in improving UWB radars’ efficiency in practice, such as post-earthquake rescue and through-wall surveillance. Besides, the potential of the operational bandwidth segmentation for target identification was demonstrated based on clustering the results from all the subsets of data. However, it was ineffective on data measured by an IR UWB radar. This was due to the spectral concentration of the IR UWB echoes, which was determined by the equivalent-time sampling architecture of the radar. This problem probably should be dealt with by an IR UWB radar with the real-time sampling architecture.

In summary, the breath detection method in the paper provides a new data processing scheme for UWB radars. With the scheme, UWB radars not only have more flexibilities in processing than narrowband radars, but also better effectiveness and efficiency than the existing UWB radars, while they are used for breath detection. So, the advanced signal processing techniques, e.g., the existing ones such as HHT, SSM and so on, can be directly included in this scheme to develop more deliberate algorithms for breath detection. Moreover, the method firstly utilizes the information provided by the ultra-wide operational bandwidth of a UWB radar. This gives us a new idea to develop algorithms to cope with other human detection problems via UWB radars. For example, analogous to the hyperspectral technology, the fine characteristics of human echoes across the whole operational bandwidth might be identified for target classification and body imaging. So further work will develop these algorithms.

## Figures and Tables

**Table 1:** Key parameters of the Multiple Input and Multiple Output (MIMO) SFCW UWB radar [25].

Parameters	Values
operational frequency	40–4400 MHz
step size	5 MHz
pulse repeated frequency (PRF)	113 Hz
unambiguous range	30 m
transmitting power	≥10 dBm
sensitivity of receiver	−90 dBm
dynamic range of receiver	≥90 dBm
